# Attentional spatial cueing of the stop-signal affects the ability to suppress behavioural responses

**DOI:** 10.1007/s00221-024-06825-8

**Published:** 2024-04-23

**Authors:** Md. Tanbeer Haque, Mariella Segreti, Valentina Giuffrida, Stefano Ferraina, Emiliano Brunamonti, Pierpaolo Pani

**Affiliations:** 1 Department of Physiology and Pharmacology, Sapienza University, Rome, Italy; 2 Behavioral Neuroscience PhD Program, Sapienza University, Rome, Italy

**Keywords:** Stop signal task, Attention, Spatial attention, Motor inhibition, Cognitive control, Executive functions

## Abstract

The ability to adapt to the environment is linked to the possibility of inhibiting inappropriate behaviours, and this ability can be enhanced by attention. Despite this premise, the scientific literature that assesses how attention can influence inhibition is still limited. This study contributes to this topic by evaluating whether spatial and moving attentional cueing can influence inhibitory control. We employed a task in which subjects viewed a vertical bar on the screen that, from a central position, moved either left or right where two circles were positioned. Subjects were asked to respond by pressing a key when the motion of the bar was interrupted close to the circle (go signal). In about 40% of the trials, following the go signal and after a variable delay, a visual target appeared in either one of the circles, requiring response inhibition (stop signal). In most of the trials the stop signal appeared on the same side as the go signal (valid condition), while in the others, it appeared on the opposite side (invalid condition). We found that spatial and moving cueing facilitates inhibitory control in the valid condition. This facilitation was observed especially for stop signals that appeared within 250ms of the presentation of the go signal, thus suggesting an involvement of exogenous attentional orienting. This work demonstrates that spatial and moving cueing can influence inhibitory control, providing a contribution to the investigation of the relationship between spatial attention and inhibitory control.

## Introduction

Our environment is constantly changing and it is crucial to remain flexible to adapt to new requests emerging in physical and social situations. Flexibility is made possible by inhibitory control, which is the ability to suppress inappropriate and non-adaptive behaviour (Wessel and Anderson 2023). Inhibitory control has been extensively investigated with the Stop Signal Task (Vince 1948; Logan and Cowan 1984; Mirabella et al. 2009; Montanari et al. 2017). In a classic version of the Stop Signal Task, participants must perform two types of randomised trials: go trials, which require a rapid response to a go signal, and stop trials, in which, after the go signal, a stop signal, which may appear unpredictably, requires the response to be inhibited. The subject’s ability to inhibit depends on the duration of the Stop Signal Delay, i.e. the time interval between the go and the stop signal (Logan and Cowan 1984; Logan 1994; Verbruggen and Logan 2008). The longer is the Stop Signal Delay, the greater is the probability of response to a stop trial. Relating the duration of the Stop Signal Delay to the probability of response it is possible to obtain the inhibition function, which is a graphical representation of the performance in the Stop Signal Task (Logan 1981; Logan and Cowan 1984). Framing the performance of this task in the Independent race model (details in methods), it is possible to obtain, together with the inhibition function, an estimate of the time it takes to inhibit the response, or the Stop Signal Reaction Time (SSRT) (Logan and Cowan 1984; Logan 1994). The SSRT has been employed as an estimation of the inhibition process in diverse studies. For instance, in neurophysiological studies this time helped in discovering the role of different brain areas in movement inhibition (Hanes et al. 1998; Schall and Hanes 1998; Paré and Hanes 2003; Chen et al. 2010; Pani et al. 2018, 2022; Giamundo et al. 2021; Brunamonti and Paré 2023); in clinical investigations it has been found that a range of pathologies are characterised by a different duration of SSRT compared to healthy controls, such as in obsessive-compulsive disorder (Mar et al. 2022), in some forms of substance use disorder (Smith et al. 2014), or in ADHD (Menghini et al. 2018; Senkowski et al. 2023). An essential consideration, particularly in the clinical application of this measure, is that the inhibition required by the Stop Signal Task comprises various processing stages, including sensory detection, option selection, and execution processes (Verbruggen et al. 2014a). This multifaceted nature allows it to be influenced by a range of factors. For example, certain aspects of the stimulus, like its luminance, its color or the sensory channel it engages, can influence the capacity for inhibition (van der Schoot et al. 2005; Montanari et al. 2017). The complexity of the task (Middlebrooks et al. 2020; Marc et al. 2023) and the reward associated with successful inhibition can also modulate it (Boehler et al. 2012, 2014; Giuffrida et al. 2023). Among the factors known to influence inhibitory control, attention can play an important role. For example, the presence of distractors can hinder the ability to inhibit (Verbruggen et al. 2014b), and suppression in a stop trial is facilitated when the stop signal follows a go signal that appears where the subject had previously attended to the go signal (Hilt and Cardellicchio 2020). Furthermore, the elimination of the fixation point affects both the initiation and inhibition of movements, ostensibly facilitating the disengagement of attention from these fixation locations (Fischer and Weber 1993; Song and Nakayama 2007; Mirabella et al. 2009). The relationship between attention and inhibition is also relevant because both functions might be supported by partially overlapping brain networks, both at the cortical and sub-cortical level (Corbetta et al. 2009; Aron et al. 2014; Alves et al. 2022). Despite these studies, the literature on the impact of attention on inhibitory control, as investigated through the Stop signal task, remains limited. In light of this, the aim of our work was to contribute to the topic by evaluating whether and how spatial cueing of attention affects inhibitory control. To achieve this we developed a stop signal task that incorporates spatial attentional modulation, drawing from previous researches (Vince 1948; Posner 1980; Logan and Cowan 1984; Abrams and Christ 2003; Smith and Abrams 2018). In this task a possible stop signal was spatially and accurately cued for most of the experiment (valid trials), while in some trials, it appeared in an uncued position (invalid trials), representing an infrequent but behaviorally relevant event. We hypothesized that in this task context, the ability to inhibit would be enhanced in valid compared to invalid trials, thus demonstrating a direct link between attentional processing and inhibitory control. This would support the view that attention is a crucial factor that must be considered when studying the ability to suppress behavioral responses (Verbruggen et al. 2014a; Leiva et al. 2015).

## Methods

### Participants

We estimated a priori the sample size of 12 subjects, on the basis of power 0.95 to detect an effect size (f = 0.57) in a within-subject design using GPower 3.1.9.7 (Faul et al. 2007, 2009), as similarly reported in a previous study by Hilt and Cardellicchio (2020) by considering the differences in stopping performance (SSRT) between valid and invalid trials.

The first inclusion criterion considered was related to a behavioural data check that is necessary to proper evaluate the ability to inhibit, by estimating the time it takes to inhibit the response, or Stop Signal Reaction Time (SSRT) in the context of the stop task. This check consists in the evaluation of the independence assumption as due on the basis of the Independent Race Model (Logan and Cowan 1984). The Independent race model, utilised to interpret performance in the Stop Signal Task, posits that two parallel processes are at play during stop trials. The first, known as the GO process, is initiated by the go signal, while the second, the STOP process, is initiated by the stop signal. The participant’s response is determined by which process wins the race: if the STOP process is faster, the response will be inhibited; if the GO process wins, the participant will respond (Logan and Cowan 1984). The model assumes that the two processes are stochastically independent, that is the timing of the GO process and the STOP process vary stochastically but without influencing each other This is the independence assumption. A prediction of this assumption is that RT in wrong stop trials, i.e. when a stop signal is presented, will be at least numerically not longer than RT in go trials (Verbruggen et al. 2019). It was assessed whether the independence between the GO process and the STOP process was met. All participants respected this criterion (see below in Data Analysis). The second inclusion criterion considered was the probability of response to the stop signal. Only participants with a probability of response to the stop signal greater than or equal to 0.25 or less than or equal to 0.75 in at least 2 Stop Signal Delays were included in the study. The probability of response to the stop signal could also meet the criteria in only one of the two experimental conditions (see below).We followed this approach because previous studies have shown that this range of probabilities allows for more reliable estimates of SSRT (Band et al. 2003; Verbuggen et al. 2019; Congdon et al. 2012). Overall, we tested 14 subjects (5 males and 9 females, mean age = 30.43 ± 11.55), but following the inclusion criteria, 2 subjects were excluded. All procedures were followed in accordance with the Declaration of Helsinki and after obtaining written informed consent from each participant. The procedure received approval from the Ethics Committee of “Roma Tre” University.

### Experimental design

The experiment was conducted using PsychoPy v.2022.2.2 software (Peirce et al. 2019), through its experiment builder. For stimuli presentation, a monitor with a resolution of 1920 × 1080 and a refresh rate of 60 Hz was used. Subjects were seated at a distance of 50–60 cm from the monitor in a darkened and sound-attenuated glass room within a larger room. The movements of the head were not restricted. One of the experimenters who collected the data monitored the subjects from outside the glass room (from the back).

In the Stop Signal Task, there are two types of trials: go and stop. In go trials, a signal prompts the subject to respond as quickly as possible. In stop trials, a go signal is initially shown, followed by a stop signal after a variable delay, known as the Stop Signal Delay. The subject must inhibit their response following the stop signal.

**Fig. 1:**
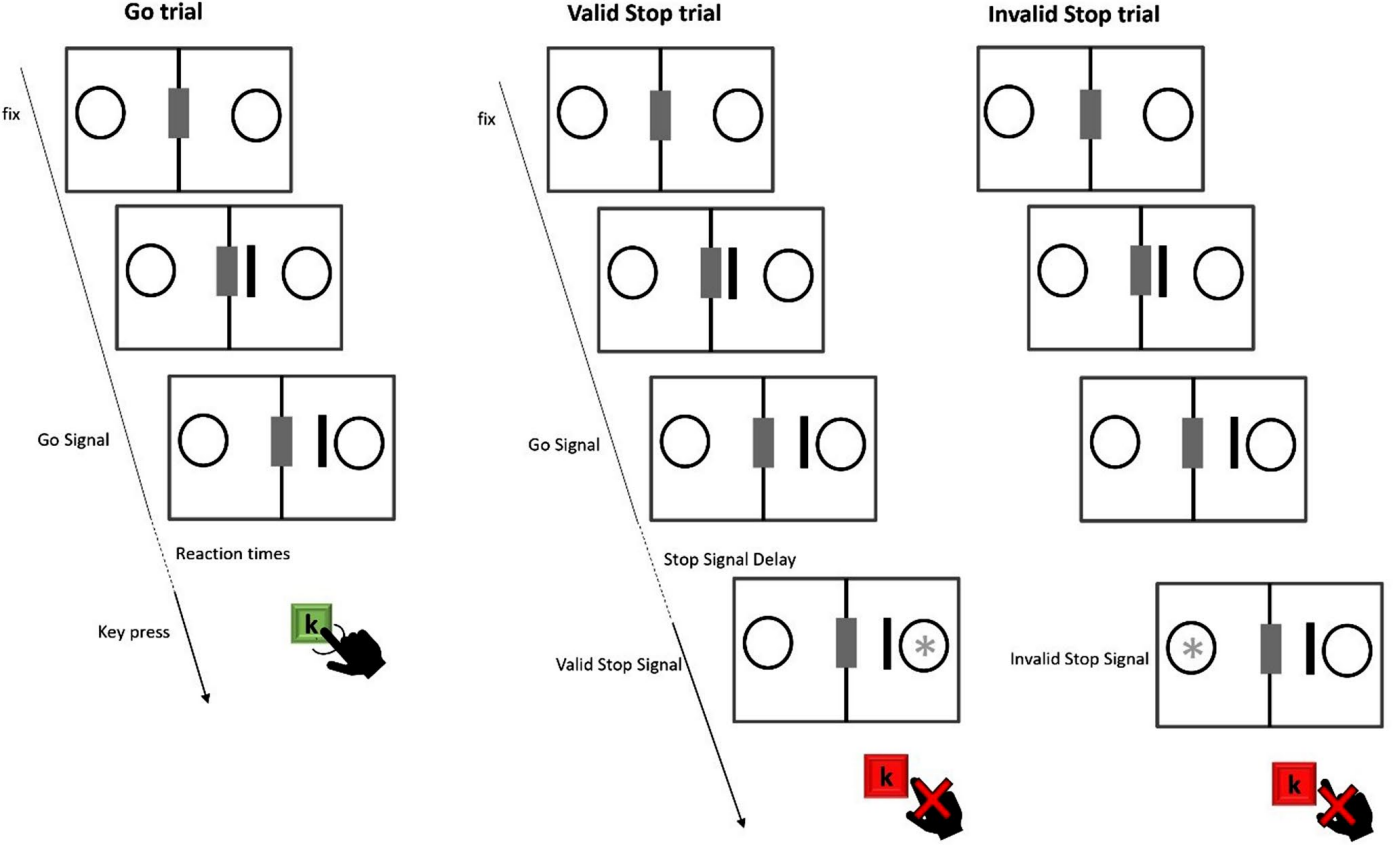
Behavioural task. Each trial starts with a central grey rectangle and two circles with a black border on a white background; then, a black bar appears and moves toward one of the two circles at intervals of 150ms. When the cue approaches the circle, it becomes a go signal. In go trials (the first trial in the figure) the subject has to by pressing the “K”. In stop trials, a stop signal (light grey asterisk) appears inside the circle after the Stop Signal Delays: in valid stop trials (the second trial in the figure), the stop signal appears in the circle reached by the bar; in invalid stop trials (the third trial in the figure), the stop signal appears in the opposite circle. The stimuli depicted in the figure are not to scale; for the actual dimensions of the scaled stimuli, please refer to the ‘experimental design’ paragraph. Only the correct trials are depicted in the figure

In our behavioural task (Fig. 1) each trial started with a screen displaying a central grey rectangle (1.3 × 5 cm) and two circles with a black border (7.45 cm of diameter) on a white background. This screen was displayed for a random duration between 800 and 1000ms. Subsequently, a black bar (the cue, 0.5 × 5 cm) appeared and moves toward one of the two circles at intervals of 150 milliseconds (the entire movement lasts 300ms) The bar moved in two steps, each covering a distance of 1.5 cm. The go signal corresponded to the cessation of movement by the bar near the circle (0.85 cm away from the circle) In go trials, the subject had to respond as fast as possible by pressing the “K” key within 800ms. In stop trials, a stop signal, represented by a light grey asterisk (1.17 cm of diameter) appeared inside the circle after the Stop Signal Delays.

In valid stop trials, the stop signal appeared in the circle reached by the bar. In invalid stop trials, the stop signal appeared in the opposite circle. We considered three Stop Signal Delays: 100ms, 250ms, and 450ms, and the number of stop trials per condition was equally divided among the 3 Stop Signal Delays. In stop trials the subject had to avoid pressing the “k” button to have a correct stop trial. The trial was wrong if the “k” button was pressed.

At the end of each trial, an auditory feedback (0.5s) indicates the performance outcome. A single beep was emitted for correctly executed trials, while two beeps were emitted for incorrectly executed trials. Participants were instructed to fix the central grey rectangle throughout the trial, respond quickly to the go signal, and try to inhibit their response when the stop signal appears in stop trials. They were also informed that the moving direction of the cue would likely predict the location of the stop signal (about 70% vs. 30%).

Before the experimental session started, participants were familiarized with the task. The experimenter first explained the task by showing the different screens, and then conducted approximately 50 familiarization trials. During these pre-test trials, the experimenter sat in front of the participants to ensure they maintained their gaze fixed on the central gray bar. All participants performed the task adequately in this phase but were not monitored during data acquisition. The trials were randomised in each block to prevent more than two consecutive stop trials. On average, subjects performed 947 go trials (59.39%), 467 valid stop trials (29.25%), and 181 invalid stop trials (11.36%), totaling about 1595 trials. These trials were divided into six blocks, all conducted in a single experimental session.

### Data analysis

Statistical analysis was implemented using MATLAB R2021b software (The MathWorks Inc., Natick, MA), in particular Statistics and Machine Learning Toolbox was also used (The MathWorks Inc., Natick, MA).

To evaluate the effects of the cueing on response inhibition, probability of response, RTs and SSRT were considered as dependent variables. Reaction times were calculated from the go signal to the time of button press. The probability of response was calculated as the ratio of the number of responses to the stop signal in a given condition (valid or invalid stop, varying Stop Signal Delay) divided by the total number of stop trials presented in that specific condition. Stop trials where the subject responded before the appearance of the go signal were excluded from these calculations. Since all subjects in our analysis adhered to the independence assumption, we estimated SSRT using the integrative method, replacing go omissions with the maximum RT (Verbruggen et al. 2019). This method entails sorting the RTs of go trials in ascending order. Then, the number of elements in the RT distribution are multiplied by the response probability of a specific Stop Signal Delay (in our case, a specific experimental condition—valid or invalid). Subtracting the specific Stop Signal Delay from the resulting nth-RT provides an estimate of SSRT. These estimates were averaged within each specific experimental condition to obtain a more reliable SSRT estimate. Consequently, SSRTs were calculated for the six stop conditions: the three Stop Signal Delays for valid stops and the three Stop Signal Delays for invalid stops, and averaged within each condition. The Wilcoxon signed-rank test (Woolson 2007) was used to test whether the independence assumption was tenable by comparing the distribution of reaction times (RTs) in go trials to that in wrong stop trials for each subject. Note that to proceed in estimating the SSRT is sufficient that RTs in wrong stop trials are numerically not longer than RTs in go trials (Verbruggen et al. 2019). To evaluate the effects of cueing across conditions, ANOVAs were performed for the SSRTs, RTs, probability of response.

## Results

### Response latencies

**Fig. 2:**
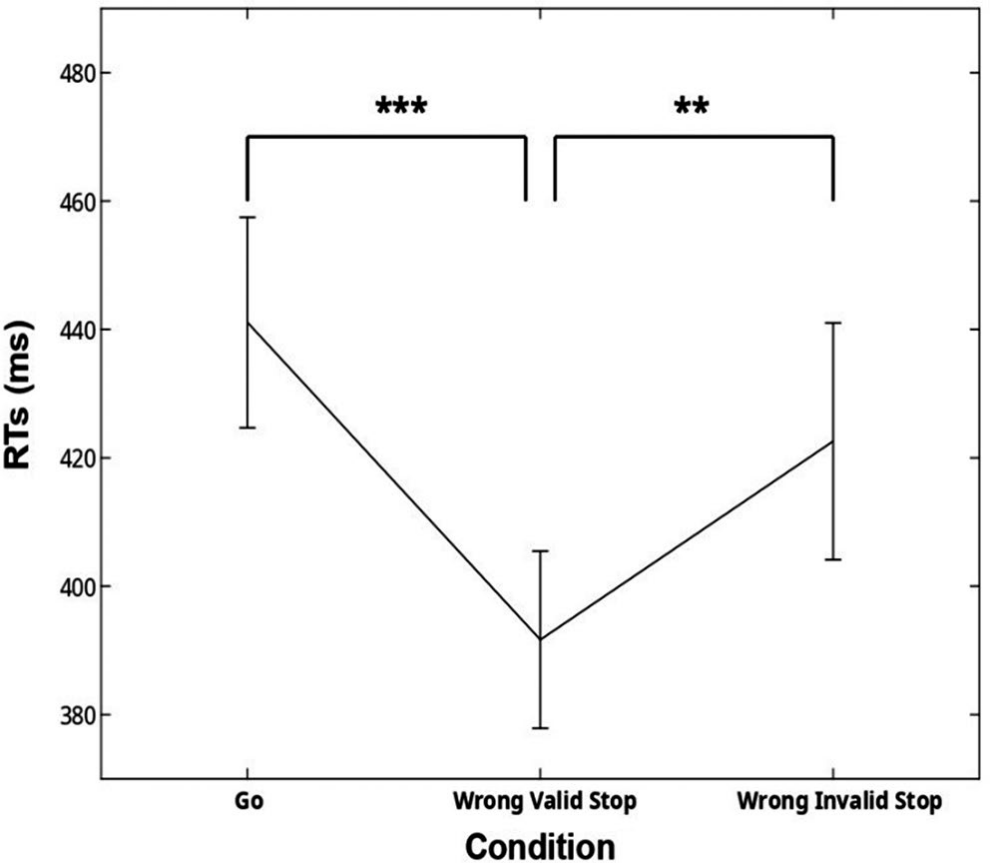
Reaction times (mean and ± 1SEM) in the three experimental conditions

We found that subject responses were numerically longer in go trials (441.08 ± 54.38ms) than in wrong valid (391.67 ± 45.81ms) and wrong invalid stop trials (422.59 ± 61.17ms, Fig. 2). These findings show that the independent assumption of the race model was respected, allowing us to estimate the SSRT. The ANOVA revealed a significant effect (F(2,20) = 10.71, *p* < .001, η_p_^2^ = 0.74). Post-hoc analysis revealed that participants were faster to respond in wrong valid stop trials than in the go and wrong invalid trials (*p* < .001 and *p* = .007, respectively).

### Cueing modulates inhibitory control at specific stop Signal Delays

**Fig. 3:**
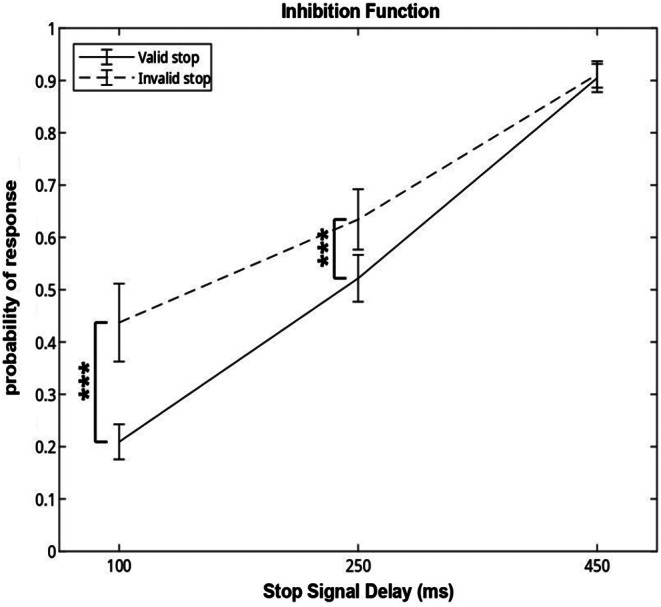
Inhibition function. Probability of response to the stop signal in the stop valid and stop invalid condition across the Stop Signal Delays

The aim of this study was to assess whether the cueing of attention influences the ability to inhibit the response. We thus evaluated the effect of the valid or invalid stop condition, and of the length of the Stop Signal Delay, on the probability of response to a stop signal. We found that the cueing affected the probability of response only at the two first delays (Fig. 3). Indeed, by running an ANOVA we found an interaction between Stop Signal Delay and Cueing (F(2,22) = 13.12, *p* < .001, η_p_^2^ = 0.54). With the Tukey-Kramer test as a post-hoc analysis, a difference was observed in the probability of response between the valid stop condition and the invalid stop condition in the first two Stop Signal Delays, while for the third the effect was not present. For both the first and second Stop Signal Delay, probability of response is significantly lower in the valid condition compared to the invalid condition (0.21 ± 0.11 vs. 0.44 ± 0.25, 0.52 ± 0.15 vs. 0.63 ± 0.19 respectively; all p’s = 0.001). The ANOVA also reported the effect of the Cueing (F(1,11) = 18.31, *p* = .001, η_p_^2^ = 0.62). Importantly, as shown in Fig. 3, the increase of Stop Signal Delay increased the probability of response in both conditions, as confirmed by the main effect (F(2,22) = 125.09, *p* < .001, η_p_^2^ = 0.92).

### Cueing modulates the length of SSRT

**Fig. 4:**
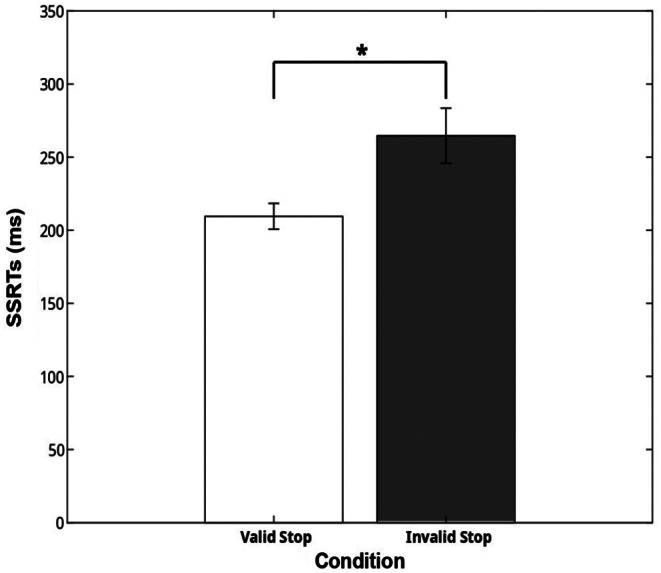
Stop signal reactiont [SSRTs (ms)] (mean and ± 1SEM) in the two stop conditions

Once we established that cueing affected the probability to inhibit we also evaluated whether this effect translated into the length of SSRT. The analysis of SSRT further supported the findings on the probability of response: the valid cueing reduces SSRT (209.5 ± 29.34ms), making this condition more effective for inhibitory control compared to the invalid condition (264.65 ± 62.53ms) (F(1,8) = 9.70, *p* = .014, η_p_^2^ = 0.54, Fig. 4).

## Discussion

This research aimed to explore the influence of spatial attention cueing on inhibitory control. Our findings indicate that spatial attention cueing increased the probability of response inhibition in the valid condition compared to the invalid condition for Stop Signal Delays ranging from 100 to 250 milliseconds. Notably, the performance remained consistent across all conditions for the maximum Stop Signal Delay (450ms). The effect of spatial cueing was further corroborated when assessing the SSRT, where the duration required to inhibit the response was observed to be shorter in the valid condition. A plausible interpretation is that the stop signal is detected more swiftly and accurately when attentional resources are already focused on its expected location (Macaluso and Doricchi 2013). In contrast, the occurrence of the stop signal in the uncued location requires an automatic reorienting of attention, elongating the inhibitory process and possibly delaying its detection. The timing of the effect’s deployment, present as early as 100ms from the Go signal and disappearing after 250ms, suggests that this effect is mostly exogenous (Mulckhuyse and Theeuwes 2010). For example, in classical studies, the use of a peripheral cue (Posner and Cohen 1984) resulted in an advantage for the cued condition that ended before 200ms, followed by the inhibition of return (Klein 2000). However, similar advantages have been observed when employing central cueing (Posner et al. 1980), and these advantages can be modulated by the predictiveness of the cues (Tipples 2002; Langdon and Smith 2005; Giessing et al. 2006; Vossel et al. 2006; Bartolomeo et al. 2007). Other factors that could have affected this effect are the circular placeholders employed. Indeed, a study reported that the presence of placeholders can facilitate cueing response if target stimuli appear shortly after the cue (100 and 200ms), while for longer target onset times, an opposite effect was reported, possibly due to the inhibition of return (Taylor et al. 2015; Klein 2000). Furthermore, the dimension of the cued area by the circles and the related attentional focus can affect processing efficiency: reducing the area is associated with a reduction in RTs (Turatto et al. 2000; Ronconi et al. 2014; Castiello and Umiltà 1992). Although we did not observe an inhibition phenomenon in our case, the presence of circles as placeholders, spatial cueing in motion, and the higher predictability of valid versus invalid stop signals could all have contributed to the effect we observed.

Previous literature has discussed how the detection phase of the stop signal can be influenced. For instance, the perceptual attributes of the stop signal can impact inhibitory control: the salience of the stop signal can modulate the SSRT in stop-signal tasks with both auditory and visual signals (van der Schoot et al. 2005; Morein-Zamir and Kingstone 2006; Camalier et al. 2007; van Gaal et al. 2009; Montanari et al. 2017). Moreover, earlier studies have demonstrated that attentional factors can affect the capacity to suppress behavioural responses: the presence of distractors negatively impacts inhibitory control (Verbruggen et al. 2014b), as does the disappearance of fixation spots, although the effect can vary depending on the motor system involved (Mirabella et al. 2009; Stevenson et al. 2009). In a recent study, participants were asked to execute a combination of an endogenous Posner task with a Stop Signal Task. In this study the stop signal was presented in the same location as the go signal (Hilt and Cardellicchio 2020). Both reaction times and SSRT were shorter in valid than invalid condition, thus demonstrating an effect of attentional allocation on the inhibitory process. A key distinction from that study and ours is that those authors assessed how reorienting for the go signal affected inhibition, thereby aligning their work with tasks that investigate the functional interaction between GO and STOP processes. Indeed in their study the cueing was primarily related to go signal, that also required a different finger response depending on the position. Thus, to be performed adequately, it required to re-orient towards the go signal and then respond to the Stop signal. It is thus possible that in that case the re-orienting required for the go signal elongates the SSRT. A series of studies have shown that the specific demands of a task can modulate a concurrent STOP process. For example, the simultaneous performance of a Stop task and Flanker or a Stroop task can negatively affect the ability to suppress a motor response (Verbruggen et al. 2004). However, this is not always the case: indeed, the modulation of the Stimulus Response Compatibility (van den Wildenberg and van der Molen 2004) or of the level of difficulty of decision making in the go task (Middlebrooks et al. 2020) and in task-switching (Verbruggen et al. 2005) do not affect stopping performance. An important difference between the Hilt and Cardellicchio (2020) and our experiment is that, in our study, the nature of valid or invalid was exclusively related to the stop signal, and that we employed fixed Stop Signal Delays, thus demonstrating that the cueing advantage tends to decrease as the Stop Signal Delay increases. Despite these differences in experimental design, Hilt and Cardellicchio (2020) found that when comparing invalid with valid trials, both RTs and SSRT were delayed in invalid trials by about 50ms, a value similar to what we observed. One could speculate that the re-orienting required for the Go signals was also affecting the inhibitory process. The reduction of the STOP process’s efficacy we observed is further supported by the longer wrong reaction times observed in the invalid conditions. This data can be readily explained by a longer STOP process that can only interrupt longer response time compared to the valid condition. Our data, however, warrants further exploration. Our research aligns with a series of studies aimed at exploring the various factors that can influence inhibitory control. Currently, it is known that inhibitory control, as measured by the Stop Signal Task, can be considered as comprising different components or stages and, in turn, being influenced by other processes (Boucher et al. 2007; Verbruggen et al. 2014a; Logan et al. 2015). Despite this, many studies using the Stop Signal Task tend to treat SSRT as the estimation of a single process. However, recently, some studies have shown, even with if with modeling methods, that deficient inhibitory capacity may be associated not so much with a generic alteration of the inhibitory process but, for example, with difficulty in detecting the stop signal and thus in triggering the necessary STOP process for inhibition (Matzke et al. 2017; Choo et al. 2022). This type of approach is particularly important for studies investigating specific neurocognitive deficits in clinical populations, where changes in the ability to inhibit can be related either to attentional or executive processes (Lampe et al. 2007; Alderson et al. 2008; Senderecka et al. 2012; Salum et al. 2014). Indeed, an inappropriate response could be due to increased distractibility or a failure to implement an adequate inhibition process (Weigard et al. 2019; Anning et al. 2023). In this context, the employment of tasks that can more precisely affect stages of cognitive processing can be helpful to disentangle diverse hypotheses or to better characterize cognitive profiles associated with specific disorders. This type of investigation would be particularly important, as often simple processing features such as processing speed or phenomena like attentional facilitation are involved in neurocognitive tasks but neglected in the definition of the functional architectures supporting performance (Salum et al. 2014). Furthermore, the possibility to influence the STOP process by means of attention can help to investigate at a high temporal definition level the neuronal dynamics supporting movement inhibition, thus helping to describe the sequence of processing that brings from the stop signal to the implementation of behavioural control, further contributing to the mechanistic understanding of neurological or neuropsychiatric pathologies (Mueller et al. 2017; Pani et al. 2022).

In this study we investigated the interplay between motor inhibition and attentional orienting. The relationship between attention and inhibition is relevant because both functions might be supported by partially overlapping brain networks, both at cortical as well sub-cortical level (Wessel and Anderson 2024; Alves et al. 2022). More specifically, motor inhibition is typically associated to a fronto-basal ganglia network, spanning different brain regions (right Inferior Frontal Gyrus or rIFG, supplementary premotor area, dorsal premotor and motor cortices, orbitofrontal cortex and Sub-thalamic nucleus (Aron and Poldrack 2006; Bryden and Roesch 2015; Diesburg and Wessel 2021; Pani et al. 2022), where the rIFG seems to play a pivotal role. The same area is implicated in attentional tasks that require reorienting to unexpected or uncued, but behaviourally relevant, stimuli, operating as part of a ventral fronto-parietal network (Bressler et al. 2008; Corbetta and Shulman 2002). In the context of the motor inhibition literature its role would be to rapidly “pause“ the ongoing planned action (Aron et al. 2014) upon the presentation of a stop signal or an unexpected event (Diesburg and Wessel 2021); in the context of the “attentional ” literature its role would be to interrupt the ongoing orienting of attention and supporting the reorienting towards the new targets, working as a “circuit breaker” (Doricchi et al. 2010; Corbetta et al. 2009). More recently, some theories have been proposed for which, inspired by the common involvement of the right inferior frontal cortex, as detected by fMRI or EEG activity, or by TMS investigations (Corbetta and Shulman 2002; Corbetta et al. 2009;Verbruggen et al. 2010; Swann et al. 2009; Hampshire et al. 2010; Lenartowicz et al. 2011) the interruption of attentional focus followed by attentional reorienting associated to any type of salient (i.e. infrequent, unexpected) event, would rely on the same “inhibitory” process at play to exert motor control (Soh and Wessel 2021; Tatz et al. 2021). As such inhibition would be at the basis of attentional re-orientating. An alternative view, the “stimulus detection account” (Leiva et al. 2015; Verbruggen et al. 2014a) has been proposed that the response to behavioural relevant unfrequent or unexpected events would require first a re-orientation of attention to detect the infrequent stimulus, and then an inhibitory response if required. Thus, while the ‘inhibitory’ theory explains the attentional shift away from the current locus towards the salient event by means of an inhibition of the current attentional representation that would also have an effect at the behavioral level, the ‘stimulus detection account’ considers the shift of attention as the ‘premise’ for properly detecting the stop signal. Our data, support the “stimulus detection account” view. Indeed, according to the ‘inhibitory’ account, the invalid stop signal, being infrequent and behaviorally relevant, should have been at least as efficient as the valid stop signal in inhibiting, as the inhibitory process would be activated first. Contrary to this prediction, we observed an elongation of SSRT in the invalid condition, which we can explain with the reorienting process necessary before implementing response inhibition. Thus, our study suggests that depending on the context, motor inhibition and attentional re-orienting can be involved at different levels in relation to the task at hand. Another aspect to consider is the role of the rIFG: the fact that this region (and possibly other brain regions) is active in different processes or tasks does not necessarily means that the function at play is always the same. This general concept has been already demonstrated by behavioral neurophysiology studies in monkeys. Indeed, in frontal eye fields some cells show neuronal modulation during saccades execution while others, colocalized with the first ones, are active in covert shifts of attention (Petersen and Posner 2012; Schafer and Moore 2007; Thompson et al. 2005). This observation demonstrates that finer-grained dissociations between functions can be detected within the same brain region, making it challenging to establish a clear connection between a specific brain region and possibly a single associated function. This consideration is further strengthened by the mixed-selectivity shown by neurons in prefrontal cortex (Rigotti et al. 2013). We recognize that in this study there are limitations. To increase the potential efficacy of the attentional cueing, we combined exogenous cueing (the movement of the bar) with predictiveness, i.e., valid trials occurred with a higher frequency (around 70%). Thus, the frequency of validity probably contributed, along with the more specific spatial cueing, to driving the effect. Further studies are needed to establish whether the effect we observed can be obtained with an equal proportion of valid/invalid or higher proportions of invalid vs. valid stop trials. A second limitation is also posed by the motion onset effect; we do not know whether a static cue could yield a similar advantage for the cued position. Therefore, it would be necessary to systematically investigate whether the effect can be evoked even with just a static exogenous cue, as done in previous papers with signal detection (Posner 1980, Posner et al. 1984). Currently, we can only say that in our case, the two characteristics are combined, leading to an advantage up to 250ms from the presentation of the go signal. Furthermore, although the difference in inhibition we observe has sizable effect, it must be confirmed in more studies.

## Data Availability

Data will be made available on reasonable request.
